# International students’ experiences on speaking and writing skills in language learning processes in higher education

**DOI:** 10.1371/journal.pone.0329331

**Published:** 2025-08-01

**Authors:** İsmail Karakuş

**Affiliations:** Classroom Teaching Department, Mersin University, Mersin, Turkey; University of Maragheh, IRAN, ISLAMIC REPUBLIC OF

## Abstract

Although speaking and writing skills play a crucial role in second language acquisition, studies focusing specifically on the challenges faced by international students learning Turkish as a foreign language remain limited. This study aims to explore the experiences of international students enrolled in higher education institutions in Turkey, particularly regarding the development of their speaking and writing skills in the context of Turkish language learning. Adopting a phenomenological design within a qualitative research framework, data were collected from 24 international students through semi-structured interviews and analyzed using content analysis. The findings revealed that students struggled with the pronunciation of certain sounds, maintaining fluency, organizing their thoughts coherently, expressing themselves accurately, applying grammar rules, and choosing appropriate vocabulary. Furthermore, participants reported experiencing anxiety about making mistakes, as well as various psychological barriers that hindered their ability to express themselves effectively. In terms of writing skills, the most prominent difficulties included grammatical errors, challenges in spelling vowels and specific consonants, misuse of punctuation, and slow writing speed. Students also indicated that their limited vocabulary and difficulties adapting to Turkish syntax negatively affected their writing performance. These findings underscore the need for integrated language instruction programs that simultaneously address speaking and writing skills, offering structured practice opportunities, anxiety-reducing strategies, and greater chances for meaningful interaction. The results provide valuable insights for language instructors, curriculum designers, and policymakers aiming to improve Turkish as a foreign language instruction tailored to the specific needs of international learners.

## Introduction

Globalization and rapid technological advancements have made education increasingly international, accelerating student mobility around the world [1]. Opportunities for intercultural and linguistic interaction have expanded significantly, further accelerating global student mobility. It can be said that these new opportunities offered by globalization and technological developments make language learning important for international students in terms of both academic success and social cohesion.

Language is a system that enables people to share their feelings, thoughts, knowledge and ideas with each other. Societies learn language to communicate and interact with each other [2]. Without language, it may not be possible for people to live together, understand and communicate. Since these students pursue their education in Turkey, it is important for international students to learn and use Turkish not only in academic fields but also in social and cultural fields [3]. Therefore, developing listening, reading, speaking, and writing skills accurately and effectively is essential. In the process of language acquisition, speaking and writing skills are considered fundamental to linguistic competence, self-expression, and effective communication [4]. While speaking skills enable individuals to express their thoughts orally in a fluent and comprehensible manner, writing skills enable them to put these thoughts into writing in a more structured and organized manner. Practicing these skills can boost learners’ self-confidence and enhance their language proficiency [5]. Moreover, speaking and writing support the internalization of grammatical structures and vocabulary through active language use [6]. In this context, the development of speaking and writing skills can play a critical role in the success of the language acquisition process. This process is also influenced by the social and emotional context in which language learning takes place. Vygotsky’s sociocultural theory emphasizes that language development is closely linked to social interaction and cultural tools, suggesting that learners acquire language more effectively through collaborative activities and scaffolded support within their social environments [7]. Similarly, Krashen’s affective filter hypothesis highlights the importance of emotional factors such as anxiety, motivation, and self-confidence in second language acquisition. A low affective filter enables better language input processing, whereas anxiety and low motivation may act as barriers to effective learning [8]. These theoretical perspectives underline the need to consider both social interaction and emotional readiness when supporting international students in developing productive language skills.

In speaking, many parameters may need to be interconnected and in harmony with each other. Since new meanings emerge during speaking, the processes are dynamic and constantly changing [9] and speaking is a complex process that may require doing many things at the same time [10]. In teaching Turkish both as a mother tongue and as a foreign language, it is important, as in every language, to pronounce the sounds correctly and to represent these sound differences properly in writing [11]. In order to acquire speaking skills, it may be necessary to know the rules of the language, i.e. grammar and pronunciation. In addition, Köksal and Pestil [12] list the characteristics of speaking skills as follows: tone and intonation, stress and rhythm, pronunciation and harmony, style, fluency, comprehensibility, syntax, accuracy, word richness, subject and sentence emphasis, completeness and appropriateness Speaking is a skill that is frequently used in education as well as in social life.

In the acquisition and development of speaking skills in the learning process, the most appropriate methods and techniques may need to be used based on the content of the subject to be covered, the level of the students, the suitability of the classroom environment, the efficiency of the activities, etc. In these activities carried out within the framework of foreign language teaching methods and techniques, the aim is to equip international students, who are learning Turkish as a foreign language, with speaking skills that will enable them to communicate effectively in Turkish [13]. An instructor who teaches speaking skills within the scope of teaching Turkish to international students may need to be good in terms of field knowledge, diction, pedagogical formation, communication, empathy, etc., as well as using body language effectively and making high use of the element of speech, which increases the effect of the word and is more important than the word. Body language, as a universal dimension of nonverbal communication, should be effectively integrated into speech instruction [14]. Considering the complexity and dynamism of the speaking process, the effective use of this skill enriches students’ language structure and vocabulary, and this accumulation can also form the basis of writing skills. This view aligns with Vygotsky’s sociocultural theory, which emphasizes the role of scaffolding provided by knowledgeable individuals, enabling learners to perform beyond their current capacities [7]. Writing skills can enable students to express their thoughts and knowledge in writing. In order to develop this skill, students can be given opportunities to work on various writing genres and grammar structures. Although the order of acquisition of language skills in foreign language learning varies, it can be said that writing skill is perceived as difficult and complex for individuals learning a new and different language. The fact that writing skill covers different skills may cause this skill to develop slowly and to be learned more difficultly [15]. Written expression can be perceived as a difficult skill due to the lack of interaction, the need for a large vocabulary and an individual dimension, which can lead to significant psychological problems in the writing process [16]. **According to Krashen, such emotional difficulties may result in a high affective filter, which hinders the internalization of language input and slows down progress in productive language skills* [*8*].* Therefore, it is very important for international students to acquire writing skills in a correct, functional and effective way not only in terms of their academic achievement but also in terms of their social and psychological processes. Writing skills can help students express their thoughts clearly and effectively. For students from different cultures, the writing process can provide an opportunity to share and express their own culture and perspectives with others. This can contribute to deepening cultural understanding [17]. Therefore, writing is an important tool for overcoming language barriers and communicating effectively across cultures [18]. Having good writing skills can increase students’ self-confidence and strengthen their self-esteem [19]. The writing process can require students to organize their thoughts, synthesize and analyze information. This process can also help them develop critical thinking skills [20]. As can be understood from these reasons, writing skill has many contributions for individuals. In order to develop this skill, activities appropriate to students’ language levels can be carried out, their motivation can be increased and their writing skills can be evaluated with comprehensive functional texts. Writing skills can be developed not only through writing activities but also by integrating reading, listening, and speaking skills. Therefore, it is important to examine the interaction of these skills within the language learning process from a holistic perspective. Developing international students’ speaking and writing skills in the language acquisition process is critical for them to communicate effectively and achieve academic success. Speaking skills can enable students to express themselves, share their thoughts and use the pragmatic aspects of language in daily life and in the classroom [21]. Writing, on the other hand, can play an important role in academic achievement by providing opportunities for deeper thinking and structuring knowledge [22]. The study of these two skills may be necessary for students to gain competence in language, develop self-confidence and learn how to use language in different contexts [5]. Moreover, developing writing and speaking skills simultaneously can reinforce students’ language knowledge and contribute to more effective use of language [23]. Environments that support creativity, personal expression, and emotional safety can contribute to lowering learners’ affective filter, thus facilitating more effective participation in writing and speaking tasks [8].

Recent studies have also emphasized the importance of integrating speaking and writing skills in Turkish as a foreign language learning. For instance, Yalçın and İnceçay [24] examined the speaking challenges of international students learning Turkish and highlighted pronunciation and fluency as major difficulties. Similarly, Aydın and Aydın [25] emphasized that international students often struggle with organizing their ideas in writing, especially due to differences in rhetorical structures across languages. Although existing studies have investigated speaking and writing skills separately, there is still a lack of comprehensive research that simultaneously examines both skills in an integrated manner within the context of Turkish as a foreign language learning. There is a limited number of studies focusing specifically on the development of speaking and writing skills in Turkish as a foreign language. Therefore, this study aims to contribute to the existing literature by addressing the experiences of international students in acquiring these skills.

When the literature is examined, there are studies on international students’ speaking skills [26–28] and writing skills [15,16,29,30]. The aim of this study is to examine the experiences of international students in the process of learning Turkish in the context of speaking and writing skills. By examining speaking and writing not in isolation but through an integrated perspective, this study offers a distinct contribution to the field by examining oral and written expression skills comprehensively and in-depth from an integrated perspective by considering the expression process as a whole. In addition, the ability of international students coming to Turkey to continue their education life in a healthy and successful way, to adapt to social life and to recognize and assimilate Turkish culture may be related to their correct, understandable and fluent use of Turkish. Therefore, this study can shed light not only for researchers and scientists but also for all stakeholders of the country such as political, social, cultural, etc.

In line with these purposes, the study aims to investigate the challenges international students face in developing their speaking and writing skills in Turkish, as well as their proposed solutions to these challenges. Accordingly, the study seeks to answer the following research questions:

1. What challenges do international students experience in developing their speaking and writing skills in Turkish language learning?
2. What solution suggestions or coping strategies do international students propose regarding these challenges?

## Research methodology

### Research design

This study, which aims to examine the experiences of international students in the process of learning Turkish in the context of speaking and writing skills, was designed in the qualitative research type. This research is in the phenomenology design, which is one of the qualitative research approaches, and in this design, it is aimed to examine the perceptions and experiences of individuals about a phenomenon and the environments and conditions that these experiences reveal [31]. Patton [32] stated that the basis of phenomenological research is the effort to understand how people interpret, perceive and understand their experiences and phenomena This approach is considered to be particularly suitable for understanding the participants’ subjective experiences about their difficulties and successes in the Turkish language. The main reasons for choosing a phenomenological design include the in-depth examination of experiences, the foregrounding of the participant perspective, and the collection of first-hand data. This design may allow individuals to describe their experiences from their own perspectives, which may increase the importance of student voice and perspective. The phenomenological approach can provide the necessary tools to answer these questions; it can facilitate the understanding of the difficulties and make the solutions proposed by the students more realistic and applicable. In line with the purpose of the study, the phenomenological approach is thought to provide an effective method for understanding the Turkish learning experiences of international students in depth and to allow both the research questions to be answered and the participants’ experiences to be evaluated more comprehensively.

### Ethical process

The ethical dimension of the research was carried out with the permission of Mersin University Social and Human Sciences Ethics Committee dated 23.09.2024 and numbered 299. The studies were conducted in accordance with local legislation and institutional requirements. Participants gave written informed consent to participate in this study. Written informed consent was obtained from the individual(s) for the publication of potentially identifiable data in this article.

### Research group

The population of the study consisted of international undergraduate students enrolled in the Departments of Turkish Education and Elementary Education (Classroom Teaching) at the Faculty of Education, Mersin University, Turkey. Since experiences are important in phenomenology studies, purposeful sampling is generally used to determine the study group [33]. In this study, purposive sampling was used, and specifically, criterion sampling — a subtype of purposive sampling — was employed to select participants who met the specific criteria of the study. Criterion sampling was used in this study because it enables the identification of problems or themes that include some differences within a group or community [32]. The main purpose of criterion sampling is to study situations that meet certain criteria predetermined by the researcher or by the structure of the research [34]. The study group of this research consists of a total of 24 international students, 14 from the Department of Turkish Education and 10 from the Department of Elementary Education (Classroom Teaching) at the Faculty of Education. Turkish department students can learn the structural and usage aspects of the language in more depth. This can enable them to better analyse their Turkish experiences. The students of the Department of Elementary Education (Classroom Teaching)department receive training especially in the initial literacy process. This is one of the most fundamental and critical stages of language teaching. As these students gain pedagogical knowledge and experience in this process, they can provide more informed feedback when assessing international students’ Turkish language skills. Moreover, their understanding of the main difficulties in language learning and their better command of strategies to cope with these difficulties can provide important insights into their Turkish language experiences. For these reasons, students from these two departments were selected. All the selected students are studying in the 4th year of undergraduate program. Because 4th year students are at the final stage of their education in the department, they may have a wider knowledge and experience in language learning and teaching. This may allow them to evaluate their Turkish language experiences more deeply and critically. They may have developed their Turkish language skills throughout their undergraduate programs and may now have reached advanced levels of the language. This may enable them to make more mature and functional evaluations of their language experiences. In addition, 4th year students usually take part in internship or practicum courses. The experiences they gain in the field of language teaching during this process may enable them to offer more practical and applicable insights into the language learning process. The criteria of the study group were that the students were selected from the 4th year of undergraduate programs in either the Department of Turkish Education or the Department of Elementary Education (Classroom Teaching)

### Data collection tool

A semi-structured interview form was used as a data collection tool in the study. While preparing the form, firstly, national and international literature was extensively reviewed and open-ended interview questions were prepared accordingly. Afterwards, in order to determine the suitability of the prepared questions for the purpose of the study, the opinions of a total of 5 field experts, 3 of whom were in the field of Turkish education, 1 in the field of the Department of Elementary Education (Classroom Teaching), and 1 in the field of Measurement and Evaluation in Education, were taken. The experts stated that the questions were suitable for the purpose of the study and that there were no problems in terms of measurement and evaluation. In order to test the comprehensibility and usefulness of the questions, a pilot study was conducted with a participant through face-to-face interviews. The pilot application was evaluated with 2 Turkish education experts and it was determined that the questions were generally understandable. When it was necessary to explain the parts that were not understood during the interview process, probing questions such as “Can you explain your opinion in more detail?” were also used [34].

### Data collection process

Participants were informed about the purpose of the study before the interview and their consent was obtained for participation in the study. It was explained to the participants that they could leave the study without any responsibility, their identity information would be kept confidential in the research results and the results would be used only for scientific purposes. The interviews were conducted individually and face-to-face by the researchers and lasted 15–30 minutes on average. The semi-structured interviews were conducted in Turkish, as all participants had sufficient proficiency in Turkish to express their experiences. In addition, the interviews were recorded with the permission of the participants. The data collection process for this study began on November 1, 2024, and ended on November 30, 2024.

### Data analysis

Content analysis method was used to analyze the data obtained from this study. First, the interview records were transcribed and the data were made ready for analysis. Inductive analysis was used in coding the data. The data were analyzed following the coding procedures proposed by Strauss and Corbin [35]. Open coding was first conducted to identify and label meaningful units of data. Then, axial coding was used to establish relationships among codes and to organize them into categories. Finally, selective coding was applied to identify core themes that represented the main findings of the study.In the analysis process of this study, separate code lists were created by two researchers simultaneously. The codes obtained were compared by the researchers and the results were integrated and categories and themes were determined. The points where the researchers differed in some coding were discussed and the codes, categories and themes were finalized by reaching consensus on common points. Since more than one researcher took part in the data analysis process, it is necessary to determine the dependability of the coding made on the same data. Yıldırım and Şimşek [34]state that inter-coder dependability in research should be at least 70%. In this study, inter-coder dependability was calculated using Miles and Huberman’s [36]dependability calculation formula (Dependability = Number of Agreements/ Number of Agreements + Number of Disagreements X 100). Coder dependability between the two researchers was calculated as 88%.

### Trustworthiness measures

In order to increase the quality of qualitative research, certain strategies are suggested by Yıldırım and Şimşek [34]. It is considered important for the credibility of the study that the research process and results are consistent, confirmable and clear. In order to ensure the credibility of this study, expert opinions were taken in the preparation of the semi-structured interview form, direct quotations were included in the findings, and the data obtained from the participants were tried to be confirmed by using probing questions [36] The transferability of qualitative research results to similar environments is possible with the transferability of the study. Thus, the research can create an understanding of similar phenomena and processes. In this study, in order to ensure transferability, the research method was explained in detail, purposive sampling method was used and the data were described in detail. The ability to look at qualitative research objectively and the researcher’s ability to act consistently throughout the research process is related to the consistency of the study. In this study, in order to ensure consistency, it was tried to act objectively and consistently throughout the research process, especially the interviews with the participants, and inter-coder dependability was calculated during the analysis process. The confirmability of the study can be explained by the fact that the conclusions reached can be continuously confirmed with the data collected and meaningful explanations can be presented to the reader within this framework. In order to ensure the confirmability of this study, the interview records were transcribed and the coding made by the coders during the analysis phase, the notes and inferences created in the reporting were kept by the researchers.

## Research results

In this section, the findings obtained by analysing the data collected related to the research questions are presented respectively.

### International students’ experiences on speaking skills in language learning processes

In this section, the answers to the question “What are the problems you experience in the process of acquiring speaking skills?” asked to international students are analyzed and the themes, categories and codes related to the answers are given in Table 1.

**Table 1:** Themes, categories and codes created for the opinions on the process of acquiring speaking skills.

Theme	Category	Code	Frequency
Articulation and Phonology Disorders	Pronunciation problems	Inability to pronounce sounds correctly	11
		Inability to pronounce vowels	5
	Expression disorders	Inability to express thoughts meaningfully	6
Grammar and Vocabulary	Disorders of verbal expression and grammar	Difficulty finding the right words	5
		Failure to understand grammar rules	2
Communication Anxiety	Self-confidence and Shyness	Hesitation in expressing oneself	5
Speech Impairment	Fluency	Lack of practice	5
		Not being able to speak fluently	3

As seen in Table 1, the most common problem reported by participants was “inability to pronounce sounds correctly” (f = 11). This was followed by “inability to pronounce vowels” (f = 5), “inability to express thoughts meaningfully” (f = 6), “difficulty finding the right words” (f = 5), “failure to understand grammar rules” (f = 2), “hesitation in expressing oneself” (f = 5), “lack of practice” (f = 5), and “not being able to speak fluently” (f = 3).

The participants stated that they could not pronounce some sounds and vowels in Turkish correctly and that they could not organize their thoughts in a meaningful way and express them correctly. They stated that they had difficulty in following grammar rules and choosing the right words when expressing their thoughts. Students stated that they were embarrassed and afraid of making mistakes while expressing themselves and that they had some psychological difficulties. They also stated that not being able to speak fluently due to lack of practice was an important problem. The following are examples of direct quotations from the students’ opinions:

Participant 4: I have a lot of difficulty in sounding out vowels. I cannot find the right words to express myself.

Participant 5: I cannot organize my thoughts in a meaningful way, that is, I cannot put them together and organize them correctly.

Participant 8: I cannot practice and therefore I cannot speak fluently.

Participant 15: I am afraid of making mistakes while speaking. Sometimes I feel very embarrassed.

The participants reported various challenges in acquiring speaking skills. Difficulties included correct pronunciation of sounds and vowels, organizing thoughts meaningfully, fluency issues due to insufficient practice, and anxiety caused by fear of making mistakes. These problems collectively hindered their ability to communicate fluently and confidently in Turkish.

### Solution suggestions regarding the problems they experienced in the process of acquiring speaking skills

In this section, the answers to the question “What are your suggestions for solutions to the problems you experience in the process of acquiring speaking skills?” asked to international students were analysed and the themes, categories and codes related to the answers are given in Table 2.

**Table 2:** Themes, Categories and Codes Related to The Solution Suggestions For The Problems Experienced İn The Process Of Acquiring Speaking Skills.

Theme	Category	Code	Frequency
Contact	Social contact and support	Chatting with Turkish friends	10
		Attending a speech course	3
	Speaking practice	Speaking lots of Turkish	10
Personal competencies	Self-confidence	Not being shy when speaking Turkish	4
Language development	Grammar and vocabulary	Learning the rules of grammar	2
		Practicing with vocabulary cards	2
Extra Activities	Read Aloud	Reading aloud	2

According to Table 2, the most frequently suggested solutions were “chatting with Turkish friends” (f = 10) and “speaking lots of Turkish” (f = 10). Other suggestions included “attending a speech course” (f = 3), “not being shy when speaking Turkish” (f = 4), “learning the rules of grammar” (f = 2), “practicing with vocabulary cards” (f = 2), and “reading aloud” (f = 2).

Participants stated that chatting with Turkish friends and attending a speaking course are important in terms of social communication and support. They stated that practicing by speaking Turkish a lot can be an important solution. It was stated that it is important not to be shy when speaking Turkish and to be more self-confident in this regard. It was stated that learning grammar rules and practicing with vocabulary cards are important for language development. It was also stated that reading aloud can also contribute to speaking skills. The following are examples of direct quotations from the students’ opinions:

Participant 9: It is necessary to speak Turkish a lot with Turkish friends.

Participant 14: It is very important to practice speaking Turkish in normal life.

Participant 23: We should not be shy when speaking Turkish. We need to be confident.

Students emphasized the importance of increasing social interaction with Turkish friends and practicing Turkish regularly in daily life. They also highlighted the need to overcome shyness, build self-confidence, and engage actively in speaking situations to improve their language skills.

### International students’ experiences on writing skills in language learning processes

In this section, the answers to the question “What are the problems you experience in the process of acquiring writing skills?” asked to international students were analyzed and the themes, categories and codes related to the answers are given in Table 3.

**Table 3:** Themes, categories and codes created for the opinions on the process of acquiring writing skills.

Theme	Category	Code	Frequency
Grammar and spelling rules	Grammar	Grammatical mistakes	7
	Spelling mistakes	Spelling of Some Sounds (Ğ and y sound)	5
		Vowels	4
	Punctuation marks	Misuse of punctuation marks	2
Writing techniques	Write Speed	Slow typing	4
Word presence	Word choice	Poor vocabulary	3
Structure of language	Syntax	Difficulty getting used to Turkish syntax	2

As presented in Table 3, participants most commonly reported “grammatical mistakes” (f = 7). This was followed by “spelling of some sounds (Ğ and Y sound)” (f = 5), “vowels” (f = 4), “misuse of punctuation marks” (f = 2), “slow typing” (f = 4), “poor vocabulary” (f = 3), and “difficulty getting used to Turkish syntax” (f = 2).

International students stated that they made many grammatical mistakes in the process of acquiring writing skills and that they had difficulty in writing some sounds such as vowels and ğ-y sounds. They stated that they use punctuation marks incorrectly and write very slowly. They stated that their vocabulary was weak and they had difficulty getting used to the syntax of Turkish. The following are examples of direct quotations regarding the students’ opinions:

Participant 15: We make too many grammatical mistakes. We have difficulty writing vowels.

Participant 18: The spelling of Turkish is different from Arabic. I mean from left to right. We could not get used to it.

Participant 23: We do not know punctuation marks, so we use them incorrectly.

International students experienced frequent grammatical and spelling mistakes, especially with Turkish vowels and specific sounds such as “ğ” and “y.” Difficulties also arose from incorrect punctuation use, slow writing speed, limited vocabulary, and challenges in adapting to Turkish syntax, which differs structurally from their native languages.

### Solution suggestions regarding the problems they experienced in the process of acquiring writing skills

In this section, the answers to the question “What are your suggestions for solutions to the problems you experience in the process of acquiring writing skills?” asked to international students were analyzed and the themes, categories and codes related to the answers are given in Table 4.

**Table 4:** Themes, categories and codes related to the solution suggestions for the problems experienced in the process of acquiring writing skills.

Theme	Category	Code	Frequency
Language development	Writing activities	Doing writing activities	13
		Keeping a diary	4
		To make composition work	2
		Writing what you listen to	2
	Grammar	Learning grammar rules	7
	Word presence	Developing vocabulary	4
Contact	Cultural and Social Interaction	Texting with friends in Turkish	3

Table 4 shows that “doing writing activities” was the most frequently mentioned solution (f = 13). Other solutions included “keeping a diary” (f = 4), “to make composition work” (f = 2), “writing what you listen to” (f = 2), “learning grammar rules” (f = 7), “developing vocabulary” (f = 4), and “texting with friends in Turkish” (f = 3).

Students stated that doing writing activities and studies can be an important solution to the problems they experience in the process of acquiring writing skills. They also stated that keeping a diary, writing compositions and writing what they listen to can also be a solution. They stated that learning grammar rules, improving vocabulary and exchanging messages with friends in Turkish can contribute to writing skills. The following are examples of direct quotations from the students’ opinions:

Participant 2: We should do a lot of writing work. We should keep a diary.

Participant 4: We need to practice composition.

Participant 6: We need to learn grammar rules.

Participant 16: We should exchange messages with our friends in Turkish, regardless of computers or phones.

The participants suggested various activities to improve writing skills, including regular writing practice, diary keeping, composition writing, and transcription exercises. They also recommended learning grammar rules, expanding vocabulary, and using written communication such as texting in Turkish to strengthen their skills.

## Discussion and conclusion

The findings of this study reveal that the difficulties international learners of Turkish as a foreign language experience in speaking and writing skills have not only linguistic but also emotional and sociocultural dimensions. In this context, in the discussion section, the prominent findings are discussed in terms of both emotional factors (anxiety, self-confidence, fear of making mistakes) and sociocultural context (interactional language acquisition, social support, cultural awareness) and deepened within the framework of existing literature and theoretical approaches.

International students reported that they had difficulty in the correct pronunciation of some sounds and vowels in Turkish and had difficulty in organizing and expressing their thoughts in a meaningful way. Similarly, Yalçın and İnceçay [24]also found that international students learning Turkish had significant difficulties with pronunciation and fluency due to phonological differences between their native languages and Turkish. These findings are in line with the literature emphasizing the difficulties created by phonetic and morphological differences in second language learning. Derwing and Munro [37]stated that correct pronunciation in a second language is a significant obstacle and this difficulty directly affects communication. In addition, Celce-Murcia et al. [38] stated that when the sound system in the second language is different from the first language, students have great difficulties in learning and using these sounds correctly.

Students expressed that they had difficulties in following grammar rules and choosing appropriate words. The fact that the participants stated that they had difficulty in following the grammar rules and choosing the right words reveals the complexity of language learning. If there are huge structural differences between the native language of a Turkish as a foreign language learner and Turkish, such as grammar rules, sentence structure, etc., the learner may make many mistakes during the conversation as he/she will transfer negatively from his/her native language. This situation may result from the fact that the learner is trapped between his/her mother tongue and the target language [39]. It may be useful to explain the similarities and differences between Turkish and their mother tongue at the beginning of speaking instruction for students learning Turkish as a foreign language. These explanations will enable students to get to know Turkish in general and to understand the other language better based on the language structures they know [27]. Ellis [40]stated that grammar and vocabulary selection in second language learning is one of the most difficult areas for students to communicate meaningfully. This situation can be solved by conducting language education in a more comprehensive and practical way. Swan and Walter [41]also stated that grammar and vocabulary selection are the most difficult areas in the learning process.

Students expressed psychological difficulties such as embarrassment and fear of making mistakes while expressing themselves. The psychological difficulties that students experience while expressing themselves can stand out as an important obstacle in the language learning process. The participants stated that they experience fear of making mistakes, anxiety and therefore embarrassment while expressing themselves. These results are also consistent with the findings of Taly and Paramasivam [42], who reported that students’ speaking anxiety stemmed from learning difficulties, cultural differences, and fear of being evaluated by peers. Emotional factors in language learning, especially speaking anxiety and self-confidence, are critical components that directly shape the individual’s learning process. According to Gardner [43] and Krashen’s [8]“Affective Filter Hypothesis”, high levels of anxiety can hinder learning attempts and make it difficult to mentally process linguistic input from the outside world. Krashen’s model proposes that emotional variables such as anxiety, motivation, and self-confidence function as a “filter” that affects the amount of input reaching the language acquisition device. When anxiety is high, this affective filter rises, preventing learners from processing input effectively. In this study, students’ reported fear of making mistakes, feelings of embarrassment, and reluctance to participate actively are clear indicators of a heightened affective filter impeding their acquisition process [8]. In this study, students’ avoidance of speaking due to fear of making mistakes and feelings of inadequacy is directly in line with Krashen’s theoretical framework.

On the other hand, Bandura’s self-efficacy theory [44]emphasizes that learners’ beliefs that they can succeed in language production determine their motivation and performance. In this context, students’ approaching the language production process with self-confidence both decreases their anxiety levels and increases their communicative competence. The majority of individuals who experience speaking anxiety think that the problem is only in themselves and worry and worry about themselves [45], which can negatively affect their self-confidence. MacIntyre and Gardner [46] stated that language anxiety reduces not only performance but also motivation to learn a language. Taly and Paramasivam [42] found that speaking anxiety stems from students’ perceptions of themselves, learning difficulties, differences between the cultures of the learners and the target language, poor language knowledge, and fear of being evaluated by peers and instructors. The strategies used to cope with speaking anxiety include various affective (relaxation), cognitive (positive thinking) and behavioral strategies (preparation/practice, avoiding eye contact, responding immediately to instructors’ questions, participating in as many speaking activities as possible, peer seeking and physical masking behavior). However, there are some studies in the literature that reach different conclusions from these findings. Sevim [47] and Solak and Yılmaz [48] concluded in their studies that international students’ Turkish speaking anxiety is low. Yashima [49]stated that some students develop various coping strategies to overcome language anxiety and that these strategies yield positive results in the language learning process. This shows that language anxiety may not always have a negative effect and in some cases it may function as a motivating factor in the learning process. Moreover, MacIntyre and Gregersen [50]stated that anxiety can have both positive and negative effects on language learning processes anxiety can motivate learners to learn a language, but it can also negatively affect their performance She emphasized that especially high levels of anxiety can reduce communication skills and self-confidence in the language learning process. However, Liu [51]in his study stated that although abnormal levels of anxiety in particular may not seem to be a good thing, normal levels of anxiety are beneficial in terms of building character, creativity, and awareness of life’s possibilities in students and that educators should explain this to students. Thus, managing anxiety can play a critical role in the learning process.

International students emphasized that not practicing enough and not being fluent is also an important problem. This problem is important in terms of showing the importance of sufficient practice in language learning. Participants emphasized that chatting with Turkish friends and attending speaking courses are important for social communication and support. They stated that practicing by speaking Turkish a lot can be an effective solution. The fact that the participants found chatting with Turkish friends and attending speaking courses important in terms of social communication and support shows that social context plays a critical role in language learning. Akay and Uzun [52]similarly emphasized that pairing international students with Turkish peers increases opportunities for authentic language practice and social integration. Language learning should be considered not only as an individual cognitive process but also as a socio-interactional activity. Vygotsky’s [7]sociocultural theory argues that language develops through social interactions, especially in the “zone of proximal development” Vygotsky emphasizes that learners achieve optimal development when they operate within their Zone of Proximal Development (ZPD), which represents the gap between what they can do alone and what they can do with support. In this zone, scaffolding provided by more competent peers or instructors facilitates learning. In the present study, international students’ preference for practicing Turkish through interaction with native peers reflects this scaffolded support, enhancing their communicative skills beyond what they could accomplish individually [7]. In this study, the students’ emphasis on chatting with their Turkish friends and their desire to practice the language in social settings support this theory. According to Swain’s [53] “output hypothesis”, an individual’s production of language, especially during social interaction, reinforces learning at both grammatical and lexical levels. In this respect, the fact that the participants emphasized the need to use Turkish frequently in social contexts clearly reveals the role of sociocultural learning environments in language development. This shows that not only in-class but also out-of-class social interactions are an integral part of the learning process. Akay and Uzun [52]concluded in their study that students wanted to be paired with Turkish students to practice speaking, unlike the instructors. This result is in line with Vygotsky’s [7]socio-cultural theory. Vygotsky argues that language learning is a social process and individuals learn language through interaction with others. Moreover, Swain and Lapkin [54]stated that language practice through social interaction in second language learning significantly facilitates language use and learning.

In addition, students stated that they should not hesitate and show more self-confidence when speaking Turkish. The fact that the students emphasized the importance of practicing by speaking Turkish a lot and that they should not hesitate in this process reveals the role of self-confidence in language learning. MacIntyre and Charos [55] stated that self-confidence increases the frequency and fluency of language use. In this context, students practicing speaking without hesitation can reduce anxiety in the language learning process and improve their language skills. Akay and Uzun [52]concluded in their study that the skill that students are most anxious about is speaking skill, and the most important reasons for speaking anxiety are the fear of making mistakes and not being able to express themselves. In developing speaking skills and reducing individuals’ anxiety, learning environments can be made flexible. Correction of mistakes can be instantaneous, the instructors’ style can be kind, empathetic and constructive, and it is also important that the instructor encourages the student to speak and increases his/her motivation.

Students stated that learning grammar rules and practicing with vocabulary cards are important for language development and that reading aloud can improve speaking skills. This can be interpreted as showing the importance of structured instruction and repetition. Nation [56] argued that repetition and structured practice in the vocabulary learning process contribute to long-term memory and support language development. In addition, students’ emphasis on improving their speaking skills by reading aloud supports efforts to increase phonological awareness. Derwing and Munro [37] stated that reading aloud and repetition help students understand the rhythm and pronunciation of language and improve their speaking skills. However, there are different views on the effectiveness of these strategies in the literature. For example, Ellis [40]argued that vocabulary cards may not always be effective because learning words without context may make it difficult for students to use these words in real life Therefore, it is suggested that contextual learning strategies should also be considered in language learning.

While speaking skills play a critical role in expressing oneself accurately and comprehensibly, strengthening writing skills, which is a more complex dimension of language learning, can be seen as important in this sense. The act of writing is a complex process that involves many cognitive, affective and psychomotor skills for students, teachers, and all individuals and has difficulties in this direction [15,57]. Studies on the academic achievement and language needs of international students show that students have the most difficulty in acquiring and developing academic writing skills [16,58,59]. This may be due to psychological, grammatical and cognitive problems experienced in the writing process [60]. In this study, international students stated that they faced various difficulties in the process of acquiring writing skills. They stated that they had problems such as making grammatical mistakes, having difficulty in writing vowel and special sounds, using punctuation marks incorrectly and having low writing speed. They also emphasized that their vocabulary was weak and they had difficulty getting used to Turkish syntax. In parallel, Azizoğlu et al. [15]and Aydın and Aydın [25] also reported that international students faced challenges in grammar, spelling, and adapting to Turkish syntax during writing tasks.

Maden et al. [57]emphasized the psychological aspect of the process by stating that students who learn Turkish as a foreign language are mostly anxious while writing and that their anxiety levels increase depending on their nationality, the alphabets they use in their countries and their reading habits. Similarly, Genç [16] also highlighted that writing anxiety is influenced by students’ limited vocabulary, lack of interaction, and individual psychological factors. Süğümlü and Bakdemir [61] stated in their study that writing lesson hours and classroom environment are inadequate in many ways, writing skill activities in textbooks are not qualified in many ways, writing activities are not sufficient and students are reluctant to write. Aydın [62]stated in his study that in Turkish writing education: Spelling and spelling-punctuation mistakes, writing words according to the local pronunciation in spoken language, inability to create sentence order and context, expression disorders such as incorrect or unnecessary use of words are the most common problems in Turkish writing education. In his study, Şimşek [63]found that international students had the most problems in the writing process, respectively: the use of affixes, sentence syntax, context-meaning errors, alphabet-related errors, the use of verbs and letter spelling errors. Research shows that individuals experience similar problems in the writing process. These problems can be solved by actively involving students in the process, using personalized learning techniques and teaching the structural and sound features of Turkish by starting from the beginner level.

Grammar errors are among the common problems encountered in the writing process. Students have difficulties in learning and applying grammar rules, which can negatively affect their writing skills. Hinkel [64], while discussing the negative effects of grammatical errors on writing skills, emphasizes the importance of grammar teaching in improving writing skills. Hinkel argues that effective teaching strategies and regular practice and feedback are necessary to minimize grammar errors. In this context, he states that a systematic approach to grammar rules plays a critical role in improving writing skills. Difficulties in writing vowels and special sounds can be considered as part of the process of getting used to the phonological structure of the language. Yılmaz [65], Kumsar and Kaplankıran [66], Çelik [67], and Işık and Işık [68] also obtained similar results in their studies by identifying that international students have problems in spelling vowels and some sounds in Turkish.

Improper use of punctuation can reduce the comprehensibility of written expressions and hinder the accurate communication of texts. Low writing speed is another problem. When slow writing is combined with grammar and spelling errors, the writing process can be very difficult and complicated for students. In this process, it is normal for students to need additional support and time and to want to practice very often. Poor vocabulary can make it difficult for students to write meaningfully and effectively. Vocabulary and vocabulary development can play an important role in improving writing skills. Difficulties in getting used to Turkish syntax reflect the difficulties students face in the process of adapting to the structural features of Turkish. Individualized teaching techniques for syntax rules and frequent practice can accelerate this adaptation process.

International students stated that various strategies were effective in improving their writing skills. They stated that methods such as writing activities and studies, keeping a diary, writing compositions, writing what they listen to, learning grammar rules, improving vocabulary and exchanging messages with friends in Turkish play an important role in this process. They stated that writing activities are an effective method in developing writing skills. Çakır [69]stated in his study that activities aiming to improve students’ writing skills should be included more in writing lessons. While keeping a diary allows students to practice language use and writing, essay writing can enable students to write more organized and detailed essays. Şimşek [63] stated in his study that in order for writing to turn into a skill, the individual should go through a long and gradual writing education process, which he listed in stages and sequentially as follows: alphabet teaching, word teaching, sentence teaching, paragraph teaching and text writing teaching. In this context, keeping a diary and doing free composition work can be a solution to the problems experienced in writing skills.

Learning grammar rules and developing vocabulary can play a critical role in strengthening writing skills. Learning grammar rules can reduce spelling and grammar errors. Vocabulary development, on the other hand, can improve writing skills by enabling students to use a wider range of words. Texting with friends in Turkish can be an effective way to practice language and improve writing skills. This method can enable students to practice daily language use and written communication.

The findings of this study also indicate that speaking and writing skills are closely interconnected in the language learning process. Difficulties in organizing thoughts during speaking, such as finding appropriate words or constructing sentences, often carry over into writing tasks, where similar challenges in vocabulary selection, sentence structure, and grammar emerge. Conversely, improvements in one skill may positively influence the other, as developing vocabulary, grammar knowledge, and confidence through speaking practice can also contribute to more coherent and accurate written expressions. This reciprocal relationship is consistent with Swain’s [53]output hypothesis, which emphasizes that both speaking and writing serve as productive modes of language output that reinforce overall language development when integrated effectively.

Based on these findings, it is recommended that language educators integrate not only grammar and vocabulary instruction but also activities that address students’ emotional and sociocultural needs. Providing frequent opportunities for meaningful speaking and writing practice, creating supportive classroom environments that reduce students’ fear of making mistakes and build their self-confidence, and offering individualized feedback may enhance both speaking and writing development. In addition, structured and diversified writing exercises such as journaling, essay writing, transcription tasks, and peer interaction through written communication can contribute to writing proficiency. Educators may also support vocabulary development, phonological awareness, and fluency-building exercises such as reading aloud and oral repetition. Implementing these strategies systematically with continuous feedback, monitoring, and evaluation can contribute significantly to international students’ language competence. These instructional strategies should be embedded within emotionally supportive environments that lower learners’ affective filter, as emphasized by Krashen [8], and should promote mediated learning through social collaboration, in line with Vygotsky’s sociocultural framework [7].

This study has several limitations that should be considered. First, the sample was limited to 24 international undergraduate students from Turkish Education and Elementary Education departments at Mersin University, which restricts the generalizability of the findings to other disciplines and institutions. Second, the study employed only qualitative data collection through semi-structured interviews; thus, quantitative or mixed-method designs were not utilized to triangulate the data. Third, the data were collected during a relatively short period, which prevents the examination of long-term language development. Finally, the participants’ native languages and cultural backgrounds may have influenced their experiences, and the findings may not fully represent international students from other linguistic and cultural contexts.

In addition, future research could expand the participant pool to include international students from diverse academic disciplines and educational levels. Longitudinal studies may also provide deeper insights into how speaking and writing skills develop over time. Moreover, experimental studies may examine the effectiveness of specific instructional interventions aimed at integrating speaking and writing activities, reducing language anxiety, and fostering students’ self-confidence and intercultural competence during Turkish language acquisition.
